# Female Firefighter Work-Related Injuries in the United States and Canada: An Overview of Survey Responses

**DOI:** 10.3389/fpubh.2022.861762

**Published:** 2022-05-09

**Authors:** Samantha Pawer, Kate Turcotte, Ediriweera Desapriya, Alex Zheng, Amanat Purewal, Alyssa Wellar, Kenneth Kunz, Len Garis, Larry S. Thomas, Ian Pike

**Affiliations:** ^1^BC Injury Research and Prevention Unit, British Columbia Children's Hospital, Vancouver, BC, Canada; ^2^Independent Medical Oncologist, and Firefighter Cancer Consultant, Victoria, BC, Canada; ^3^School of Criminology and Criminal Justice, University of the Fraser Valley, Abbotsford, BC, Canada; ^4^Surrey Fire Service, City of Surrey, Surrey, BC, Canada; ^5^Department of Pediatrics, The University of British Columbia, Vancouver, BC, Canada

**Keywords:** women's health, firefighters, occupational injuries, risk factors for injury, survey

## Abstract

**Objectives:**

This study explored how demographic characteristics, life experiences, and firefighting experiences have an impact on work-related injuries among female firefighters, and described events surrounding such work-related injuries.

**Methods:**

This online survey was available from June 2019 to July 2020. Questions related to demographic characteristics, life experiences, firefighting experiences, and work-related injuries. Descriptive analyses characterized variables by the presence or absence of work-related injury, injury severity, job assignment, and country of residence.

**Results:**

There were 1,160 active female firefighter survey respondents from the US and Canada, 64% of whom reported having at least one work-related injury. US respondents made up 67% of the total but 75% of the injured sample. Injured respondents were older, had been in the fire service longer, and had a greater number of fires and toxic exposures than non-injured respondents. Heavier weight, tobacco use, and alcohol consumption were more common among injured respondents. The two most common contributing factors to work-related injuries were human error and firefighter fatigue. Among respondents who reported an injury-related time loss claim, 69% were wearing protective equipment when injured, and 9% of the injuries directly resulted in new policy implementation.

**Conclusions:**

These findings can help inform resource allocation, and development of new policies and safety protocols, to reduce the number of work-related injuries among female firefighters.

## Introduction

Firefighters are essential workers who respond to fires, vehicle collisions, medical emergencies, hazardous material spills, rescues, and other potentially harmful events. Such critical incidents can have negative consequences for individual and organizational firefighting performance (1). This type of work can cause declines in the physical and mental health of firefighters, and thus their ability to work (2). In the United States (US), firefighters experienced an estimated >30,000 fire ground injuries per year from 2010 to 2014, with similar rates of 61.6 per 1,000 male and 61.2 per 1,000 female firefighters (3). US emergency departments treated ~351,800 injured firefighters from 2003 to 2014, 95% of whom were males (4).

While males make up approximately half of the general population in the US and Canada (5, 6), they reflect the majority among the general firefighting population in the existing literature. In 2018, females accounted for ~8% of all US firefighters, and just 4% of career firefighters (7), with similar trends observed in Canada (6). Although only a small proportion, between the US and Canada collectively, there are ~95,145 female firefighters: 93,700 in the US (career and volunteer) (7), and 1,445 in Canada (career only) (8). Yet, little is known about this sub-population, particularly when it comes to the details regarding work-related injuries.

While many firefighter injury studies do not exclude females, females typically make up such a small proportion of samples that it is difficult to draw any specific conclusions regarding their health outcomes (9, 10). Furthermore, these studies often do not analyze data by sex or gender (4, 11). In an international survey of 840 female firefighters in 14 countries, those in North America were more likely to have been injured than those in the UK and Ireland, Australasia, and Europe (12). However, this study focused on health and wellbeing in general, and did not provide details regarding injury events. Of 3,012 female firefighters surveyed in the US, 31.7% reported at least one work-related injury within the past year, 43% of which resulted in work loss and 25% of which became chronic injuries, like back pain (13).

More information is needed in order to inform best safety practices and injury prevention policies. The present study was uniquely designed to address existing knowledge gaps by collecting comprehensive descriptions about work-related injuries among female firefighters. The objectives of this study were to: (1) describe the demographic characteristics and life experiences of female firefighters; (2) describe firefighting experiences of female firefighters; and (3) describe events resulting in work-related injuries among female firefighters and the types of injuries sustained. The conclusions from this study will be important for developing and evaluating health and wellness policies, designating resources, and designing surveillance, education, and prevention strategies to make the workplace safer for, and more supportive of, female firefighters.

## Methods

### Study Design and Recruitment

An online cross-sectional survey was developed by the research team and distributed globally to collect information from career and volunteer female firefighters, as approved by the University of British Columbia Children's and Women's Research Ethics Board (H18-03318). The full survey was professionally translated into French by British Columbia Professional Legal Interpreters, Inc. The survey was advertised on industry-specific magazine sites and blogs promoting firefighter safety and health, including, Firefighting in Canada, and Fire and EMS Leader Pro. Additionally, participants were recruited via postings and email lists by agencies including: the International Association of Firefighters; CTIF—The International Association of Fire & Rescue Services; Center for Fire, Rescue and EMS Health Research at the National Department of Research Institutes; Fire and Rescue New South Wales; and the Memphis Fire Department. Snowball sampling was employed, which is a non-probability sampling technique where existing subjects recruit future subjects from among their acquaintances (14).

Prospective participants were directed to the online survey to learn more about the study, before consenting to participate. Using REDCap electronic data capture tools hosted at the Provincial Health Services Authority (15, 16), the online survey was available in both English and French from June 7, 2019 to July 19, 2020.

### Survey Content

The survey contained 85 questions. The Demographic Profile included information on sex/gender, age, height, weight, country of residence, and ethnicity. The Lifestyle Profile included history of tobacco use and alcohol consumption. The Firefighter Profile included questions about time in the fire service (e.g., years since starting and/or leaving the service), department type and rank, firefighting experiences including estimated number of career fires and toxic exposures, and whether the respondent had Presumptive Coverage for specified injuries or diseases presumed to be work-related. The Injury Profile focused on work-related injuries: injury type, severity, body part, event during injury (e.g., fire ground, in-transit, training), contributing factors (e.g., equipment failure, lack of training, firefighter fatigue), time off and return to work, protective equipment at time of injury, and policies instituted as a direct result of the injury. In addition, the Cancer Profile included questions about cancer and pre-cancer experiences (e.g., type of cancer/pre-cancer, stage and grade, method of detection and treatment, family history), with the additional lifestyle variables of hormone use, pregnancy, and breast feeding, all of which will be reported in a separate paper.

Survey questions were primarily multiple choice with pre-selected options (as seen in the results); many allowed multiple selections per question. Qualitative analyses of the open-ended questions will be reported in a separate paper. The survey was branched, with some questions only available based on answers to previous questions.

### Exclusion Criteria and Data Cleaning

Upon survey closure, data from all 1,723 entries were extracted. Entries were removed if all questions were left blank, if it was a duplicate entry from the same respondent, or if the respondent reported that they were both born as and identify as male, leaving 1,344 surveys. We aimed to have an inclusive sample, including anyone born female (regardless of how they currently identify) or who was born male but currently identify as female. Due to low numbers from some countries, those making up fewer than 5% (67 respondents) of this sample were excluded (e.g., Australia, New Zealand, the UK) in order to provide a more stable description of injury among female firefighters without issues with geographical differences in firefighting or lifestyle experiences, leaving 1,232 responses from the US and Canada. Responses from 72 inactive firefighters were excluded to ensure results reflect current female firefighters and their experiences with work-related injuries. The final sample size was 1,160 respondents.

### Data Analysis

Descriptive analyses were conducted on the final dataset of active female firefighters residing in the US and Canada. Survey response rates were calculated using the number of female firefighters published for the US (7) and Canada (17) (assuming 4.4% of Canadian firefighters are female, as reported by Statistics Canada) (8). Demographic characteristics, life experiences, and firefighting experiences were described by the presence or absence of a work-related injury. These data were further broken down by injury type and severity, with groups including injuries requiring <2 weeks off work (reported only, received first aid only, treated by a doctor only, those who missed <2 weeks), 2–4 weeks off work, more than 4 weeks off work, and life-threatening injuries. Firefighting experiences were assessed for injured and non-injured samples by country of residence. Characteristics of work-related injuries were broken down by department (career, volunteer, both), and country of residence. Continuous data were presented as means and standard deviation for normal data, and as medians and quartile distributions for non-normal data, while categorical data were displayed as counts with an associated proportion of the sample. Continuous variables for the US and Canada were compared using two-sample *t*-tests for normal data and Wilcoxon rank-sum tests for non-normal data, and categorical variable responses were compared using Chi-square (χ^2^) tests, to a significance level of *p* ≤ 0.05.

## Results

### Demographics and Life Experiences

A total of 1,160 active female firefighters from the US (67%) and Canada (33%) were included in the analyses (Table 1). Our sample represents ~0.8% of US and 5.8% of Canadian female firefighters. Overall, 99% of the sample were individuals both born female and currently identifying as female, and the mean age was 40.5 years (SD = 9.6, range = 18–71). In total, 64% of respondents experienced at least one work-related injury, including 554 (72%) among the US respondents and 188 (48%) among the Canadian respondents. Injuries resulting in fewer than 2-weeks of lost work time were the most common at 64% of all injuries, followed by those resulting in more than 4-weeks of lost work (38%), 2- to 4-weeks of lost work (14%), and life-threatening injuries (2%). Three quarters (*n* = 554) of all injuries were among respondents in the US. This proportion of US respondents increases with higher injury severity from 71% of those with <2-weeks work lost, up to 87% with life threatening injuries.

**Table 1:** Demographic characteristics of active female firefighter survey respondents in the US and Canada, by injury severity as measured by time of work lost.

	**Entire sample (*n* = 1,160)**	**All injuries** **(*n* = 742)**	**No injuries** **(*n* = 418)**	** <2 weeks work lost^a^ (*n* = 494)**	**2–4 weeks work lost (*n* = 106)**	**>4 weeks work lost (*n* = 285)**	**Life threatening** **(*n* = 15)**
	***N*** **(%)**	***N*** **(%)**	***N*** **(%)**	***N*** **(%)**	***N*** **(%)**	***N*** **(%)**	***N*** **(%)**
**Country of residence**							
USA	772 (67)	554 (75)	218 (52)	353 (71)	77 (73)	238 (84)	13 (87)
Canada	388 (33)	188 (25)	200 (48)	141 (29)	29 (27)	47 (16)	^*^
**Sex, gender**							
Female, female	1153 (99)	736 (99)	417 (99)	488 (99)	106 (100)	285 (100)	15 (100)
Female, male	0 (0)	0 (0)	0 (0)	0 (0)	0 (0)	0 (0)	0 (0)
Female, non-binary	^*^	^*^	0 (0)	^*^	0 (0)	0 (0)	0 (0)
Male, female	^*^	^*^	^*^	^*^	0 (0)	0 (0)	0 (0)
	**Mean (SD)**	**Mean (SD)**	**Mean (SD)**	**Mean (SD)**	**Mean (SD)**	**Mean (SD)**	**Mean (SD)**
Age (years)	40.5 (9.6)	42.5 (9.1)	36.9 (9.3)	41.7 (9.3)	45.2 (7.3)	44.9 (8.7)	50.4 (7.4)
Height (cm)	168.4 (7.5)	168.5 (7.2)	168.0 (6.4)	168.3 (7.0)	169.2 (6.5)	168.7 (7.1)	167.8 (7.3)
Weight (kg)	74.1 (13.6)	75.7 (14.2)	71.4 (11.5)	74.3 (13.1)	77.3 (14.8)	78.4 (15.0)	72.4 (12.6)
Body Mass Index (BMI)	26.1 (4.6)	26.7 (4.8)	25.2 (4.1)	26.3 (4.4)	27.0 (5.2)	27.6 (4.9)	25.5 (3.0)
	***N*** **(%)**	***N*** **(%)**	***N*** **(%)**	***N*** **(%)**	***N*** **(%)**	***N*** **(%)**	***N*** **(%)**
Used tobacco	289 (25)	196 (26)	93 (22)	126 (26)	20 (19)	78 (27)	^*^
Consumed alcohol	1,057 (91)	690 (93)	367 (88)	466 (94)	101 (95)	259 (91)	13 (87)

a *Includes injuries that were reported only, received first aid only, treated by a doctor only, and those that missed <2 weeks of work*.

* *Fewer than 5 respondents*.

Injured respondents tended to be older and heavier (at the time of survey response) than those who were not injured (Table 1). There was no relationship found between age and body mass index (BMI) at the time of survey response (*r*^2^ = 0.004). Injured respondents had slightly higher proportions of tobacco use (26%) and alcohol consumption (93%), as compared to those who had never been injured.

### Firefighting Experience

Greater length of time in the fire service, number of career fires, and number of toxic exposures generally increased with injury severity (Table 2). Career firefighters were injured more than volunteers. By rank, firefighters made up a smaller proportion of the sample as injury severity increased, while company/station officers made up larger proportions with increasing severity. Most recent assignment did not differ between injured and non-injured respondents. A higher proportion of injured respondents took a leave of absence than non-injured.

**Table 2:** Firefighting experience of active female firefighter survey respondents in the US and Canada, by injury severity as measured by time of work lost.

	**Entire sample (*n* = 1,160)**	**All injuries** **(*n* = 742)**	**No injuries** **(*n* = 418)**	**<2 weeks work lost^a^ (*n* = 494)**	**2–4 weeks work lost (*n* = 106)**	**>4 weeks work lost (*n* = 285)**	**Life threatening** **(*n* = 15)**
	**Median** **(Q1, Q3)**	**Median** **(Q1, Q3)**	**Median** **(Q1, Q3)**	**Median (Q1, Q3)**	**Median (Q1, Q3)**	**Median (Q1, Q3)**	**Median (Q1, Q3)**
Years in fire service	13 (5, 20)	16 (9, 22)	7 (3, 15)	15 (7, 21)	19 (12, 24)	19 (12, 24)	28 (21, 32)
Estimated career fires^b^	40 (15, 100)	50 (20, 200)	20 (8, 50)	50 (20, 150)	80 (30, 200)	80 (25, 200)	200 (80, 600)
Estimated toxic exposures^b^	5 (1, 20)	10 (2, 30)	3 (0, 10)	10 (2, 25)	10 (2, 50)	10 (2, 50)	25 (10, 200)
	***N*** **(%)**	***N*** **(%)**	***N*** **(%)**	***N*** **(%)**	***N*** **(%)**	***N*** **(%)**	***N*** **(%)**
**Department**							
Career	824 (71)	569 (77)	255 (61)	377 (76)	86 (81)	225 (79)	13 (87)
Career/volunteer	220 (19)	137 (18)	83 (20)	91 (18)	15 (14)	51 (18)	0 (0)
Volunteer	83 (7)	27 (4)	56 (13)	19 (4)	^*^	7 (2)	^*^
Other	33 (3)	9 (1)	24 (6)	7 (1)	^*^	^*^	0 (0)
**Most recent rank** ^c^							
Firefighter	757 (65)	435 (59)	322 (77)	300 (61)	56 (53)	141 (49)	^*^
Paramedic	270 (23)	201 (27)	69 (17)	129 (26)	37 (35)	83 (29)	^*^
Company/Station officer	231 (20)	179 (24)	52 (12)	121 (24)	33 (31)	93 (33)	9 (60)
Driver/Engineer	191 (16)	142 (19)	49 (12)	92 (19)	22 (21)	58 (20)	^*^
EMT	188 (16)	100 (13)	88 (21)	72 (15)	7 (7)	34 (12)	^*^
Chief officer/Superintendent	81 (7)	59 (8)	22 (5)	37 (7)	7 (7)	21 (7)	^*^
Master technician	8 (1)	8 (1)	0 (0)	6 (1)	^*^	^*^	0 (0)
Other	95 (8)	54 (7)	41 (10)	33 (7)	12 (11)	18 (6)	^*^
**Most recent assignment**							
Engine	615 (53)	392 (53)	223 (53)	265 (54)	56 (53)	149 (52)	5 (33)
Truck (Ladder/Aerial)	98 (8)	63 (8)	35 (8)	42 (9)	8 (8)	20 (7)	^*^
EMS unit (Ambulance)	97 (8)	66 (9)	31 (7)	40 (8)	6 (6)	28 (10)	0 (0)
Special operations/Rescue	43 (4)	24 (3)	19 (5)	19 (4)	^*^	7 (2)	0 (0)
HazMat unit	14 (1)	9 (1)	5 (1)	5 (1)	^*^	^*^	^*^
Other	291 (25)	188 (25)	103 (25)	123 (25)	32 (30)	78 (27)	8 (53)
Took leave of absence	327 (28)	236 (32)	91 (22)	151 (31)	34 (32)	106 (37)	7 (47)

a *Includes injuries that were reported only, received first aid only, treated by a doctor only, and those that missed <2 weeks of work*.

b *Responses over 16,000 were excluded*.

c *Questions in which participants could select multiple answers*.

* *Fewer than 5 respondents*.

Respondents in the US reported a median average of 15-years (Quartiles: 7, 22) in the fire service, compared to 10-years (Quartiles: 4, 17) reported by respondents in Canada (Table 3). US respondents injured on the job tended to have a higher number of career fires than those in Canada, with a median average of 40 (Quartiles: 15, 150) vs. 30 (Quartiles: 15, 80), and both countries had many fewer career fires among those who had not been injured. A similar pattern was seen for toxic exposures. Conversely, a higher proportion of respondents in Canada reported taking a leave of absence than those in the US. There was little difference between respondents in the US and Canada by department, rank, or most recent assignment.

**Table 3:** Firefighting experience of active female firefighter survey respondents in the US and Canada, by country and injury status.

	**Entire US sample (*n* = 772)**	**All injuries in the US (*n* = 554)**	**No injuries in the US (*n* = 218)**	**Entire Canada sample (*n* = 388)**	**All injuries in Canada (*n* = 188)**	**No injuries in Canada (*n* = 200)**
	**Mean (SD) or Median (Q1, Q3)**	**Mean (SD) or Median (Q1, Q3)**	**Mean (SD) or Median (Q1, Q3)**	**Mean (SD) or Median (Q1, Q3)**	**Mean (SD) or Median (Q1, Q3)**	**Mean (SD) or Median (Q1, Q3)**
Age	41.1 (9.5)	42.8 (9.2)	37.0 (8.9)	39.2 (9.6)	41.8 (8.7)	36.8 (9.8)
BMI	26.5 (4.8)	26.9 (5.0)	25.3 (3.9)	24.4 (4.1)	26.0 (3.9)	25.1 (4.3)
Years in fire service	15 (7, 22)	17 (10, 23)	8 (3, 17)	10 (4, 17)	12.5 (6, 18)	6 (3, 13)
Estimated career fires^a^	40 (15, 150)	60 (20, 200)	20 (10, 50)	30 (15, 80)	50 (20, 100)	20 (7, 50)
Estimated toxic exposures^a^	6 (1.5, 27.5)	10 (2, 50)	4 (0, 10)	4 (1, 14)	5 (2, 20)	2 (0, 10)
	***N*** **(%)**	***N*** **(%)**	***N*** **(%)**	***N*** **(%)**	***N*** **(%)**	***N*** **(%)**
Took leave of absence	204 (26)	162 (29)	42 (19)	123 (32)	74 (39)	49 (25)
**Department**						
Career	593 (77)	437 (79)	156 (72)	231 (60)	132 (70)	99 (50)
Career/Volunteer	151 (20)	106 (19)	45 (21)	69 (18)	31 (16)	38 (19)
Volunteer	19 (2)	7 (1)	12 (6)	64 (16)	20 (11)	44 (22)
Other	9 (1)	^*^	5 (2)	24 (6)	5 (3)	19 (1)
**Most recent rank** ^b^						
Firefighter	453 (59)	292 (53)	161 (74)	304 (78)	143 (76)	161 (81)
Paramedic	250 (32)	187 (34)	63 (29)	20 (5)	14 (7)	6 (3)
Company/Station officer	172 (22)	142 (26)	30 (14)	59 (15)	37 (20)	22 (11)
Driver/Engineer	146 (19)	111 (20)	35 (16)	45 (12)	31 (16)	14 (7)
EMT	172 (22)	92 (17)	80 (37)	16 (4)	8 (4)	8 (4)
Chief officer/Superintendent	68 (9)	54 (10)	14 (6)	13 (3)	5 (3)	8 (4)
Master technician	6 (1)	0 (0)	6 (3)	^*^	0 (0)	^*^
Other	43 (6)	29 (5)	14 (6)	52 (13)	25 (13)	27 (14)
**Most recent assignment**						
Engine	423 (55)	294 (53)	129 (59)	192 (49)	98 (52)	94 (47)
Truck (Ladder/Aerial)	59 (8)	46 (8)	13 (6)	39 (10)	17 (9)	22 (11)
EMS unit (Ambulance)	89 (12)	60 (11)	29 (13)	8 (2)	6 (3)	^*^
Special operations/Rescue	12 (2)	10 (2)	^*^	31 (8)	14 (7)	17 (9)
HazMat unit	9 (1)	6 (1)	^*^	5 (1)	^*^	^*^
Other	180 (23)	138 (25)	42 (19)	111 (29)	50 (27)	61 (31)

a *Responses over 16,000 were excluded*.

b *Questions in which participants could select multiple answers*.

* *Fewer than 5 respondents*.

### Work-Related Injuries

Career firefighters were injured more often than volunteers; 69% (*n* = 569) of career firefighters were injured, while 33% (*n* = 27) of volunteers were injured (Table 4). Among career, career/volunteer, and volunteer female firefighters, the most common injury types were strains, sprains and muscle pain, while extremities, backs, shoulders/chests were the most commonly injured body parts. Leading contributing factors for work-related injuries were human error, fatigue, and lack of situational awareness. Upon returning to work after an injury time-loss claim, 35% (*n* = 118) of female firefighters had modified duties, 29% (*n* = 100) had graduated return-to-work processes, and 18% (*n* = 62) had other supports. As well, in 9% (*n* = 30) of cases, a new policy was made directly resulting from the injury.

**Table 4:** Characteristics of work-related injuries among active female firefighters in the US and Canada, by department.

	**Entire sample** **(*n* = 1,160)**	**Career** **(*n* = 824)**	**Career/Volunteer** **(*n* = 220)**	**Volunteer** **(*n* = 83)**
	**Median (Q1, Q3)**	**Median (Q1, Q3)**	**Median (Q1, Q3)**	**Median (Q1, Q3)**
Number time loss claims/respondent	0 (0, 1)	0 (0, 1)	0 (0,1)	0 (0, 0)
Average time loss/injured respondent (months)	1 (0.5, 3)	1.3 (0.5, 4)	1 (0.4, 3.5)	1 (0.6, 2)
	***N*** **(%)**	***N*** **(%)**	***N*** **(%)**	***N*** **(%)**
Injured at work	742 (64)	569 (69)	137 (62)	27 (33)
**Injury severity** *^a^*				
Reported only	242 (21)	192 (23)	40 (18)	8 (10)
First aid only	170 (15)	129 (16)	33 (15)	6 (7)
Treated by doctor only	249 (21)	192 (23)	48 (22)	8 (10)
<2 weeks lost work	182 (16)	143 (17)	33 (15)	^*^
2–4 weeks lost work	106 (9)	86 (10)	15 (7)	^*^
>4 weeks lost work	285 (25)	225 (27)	51 (23)	7 (8)
Life-threatening injury	15 (1)	13 (2)	0 (0)	^*^
Injury time loss claim (*n* = 741, 569, 136, 27)	339 (46)	265 (47)	61 (45)	8 (30)
Returned to work	318 (94)	247 (93)	59 (97)	8 (100)
	**All injury time-loss claims (*****n*** **= 339)**	**Career injury time-loss claims (*****n*** **= 265)**	**Career/Volunteer injury time-loss claims (*****n*** **= 61)**	**Volunteer injury time-loss claims (*****n*** **= 8)**
	***N*** **(%)**	***N*** **(%)**	***N*** **(%)**	***N*** **(%)**
**Event during injury**				
Fire ground	97 (29)	78 (29)	12 (20)	^*^
Other on-duty	93 (27)	75 (28)	15 (25)	^*^
Training	68 (20)	49 (18)	18 (30)	^*^
Non-fire emergency	63 (19)	52 (20)	9 (15)	^*^
In-transit	18 (5)	11 (4)	7 (11)	0 (0)
Protective equipment worn while injured	233 (69)	182 (69)	41 (67)	6 (80)
Pre-existing condition contributed to injury	26 (8)	20 (8)	5 (8)	0 (0)
**Injury type** ^a^				
Strain/sprain/muscle pain	188 (55)	138 (52)	41 (67)	7 (90)
Dislocation/fracture	75 (22)	64 (24)	10 (16)	0 (0)
Wound/cut/bleeding/bruise	30 (9)	23 (9)	6 (10)	0 (0)
Other respiratory distress	8 (2)	5 (2)	^*^	0 (0)
Eye injury	7 (2)	5 (2)	^*^	0 (0)
Smoke/gas inhalation	6 (2)	5 (2)	^*^	0 (0)
Thermal stress	^*^	^*^	^*^	0 (0)
Fire/chemical burn	^*^	^*^	^*^	0 (0)
Act of violence	^*^	^*^	^*^	^*^
Other injury	93 (27)	75 (28)	15 (25)	^*^
**Injured body part** ^a^				
Extremity	94 (28)	75 (28)	15 (25)	^*^
Back	83 (24)	64 (24)	16 (26)	^*^
Shoulder/chest	68 (20)	53 (20)	14 (23)	0 (0)
Hand	42 (12)	34 (13)	7 (11)	^*^
Foot	31 (9)	24 (9)	6 (20)	^*^
Head/face	25 (7)	17 (6)	6 (10)	^*^
Abdomen	6 (2)	5 (2)	^*^	0 (0)
Other	51 (15)	42 (16)	7 (11)	^*^
**Contributing factor** ^a^				
Human error	76 (22)	60 (23)	12 (20)	^*^
Firefighter fatigue	34 (10)	24 (9)	8 (13)	^*^
Lack of situational awareness	31 (9)	24 (9)	6 (10)	0 (0)
Weather/act of nature	30 (9)	24 (9)	5 (8)	^*^
Decision making	28 (8)	25 (9)	^*^	0 (0)
Equipment failure	25 (7)	19 (7)	6 (10)	0 (0)
Lack of communication	22 (6)	18 (7)	^*^	^*^
Crew size	17 (5)	14 (5)	^*^	^*^
Lack of wellness/fitness	16 (5)	12 (5)	^*^	0 (0)
Lack of teamwork	14 (4)	11 (4)	^*^	0 (0)
Officer/incident command	11 (3)	10 (4)	^*^	0 (0)
Dangerous substance	9 (3)	7 (3)	^*^	0 (0)
Civilian error	8 (2)	5 (2)	^*^	0 (0)
Lack of training	8 (2)	8 (3)	0 (0)	0 (0)
Structural failure	7 (2)	7 (3)	0 (0)	0 (0)
Act of violence	6 (2)	^*^	^*^	^*^
Standard operating guideline/procedure breach	5 (1)	^*^	^*^	0 (0)
Protective equipment not worn	^*^	^*^	^*^	0 (0)
Protocol breach	^*^	^*^	0 (0)	0 (0)
Horseplay	^*^	^*^	0 (0)	0 (0)
**Return to work schedule** ^a^				
Modified duties	118 (35)	93 (35)	21 (34)	^*^
Graduated return	100 (29)	72 (27)	21 (34)	^*^
Other supports	62 (18)	48 (18)	13 (21)	^*^
New policy made directly resulting from injury	30 (9)	23 (9)	5 (8)	^*^

a *Questions in which participants could select multiple answers*.

* *Fewer than 5 respondents*.

Per respondent, female firefighters in the US reported significantly more injury time loss claims than in Canada (*p* = 0.015), and significantly longer length of time loss per injured respondent (*p* = 0.012; Table 5). A significantly higher proportion of respondents from the US reported being injured at work (*p* < 0.001), with different injury severity profiles (*p* = 0.012). The most common injury severity among respondents in the US was missing more than 4-weeks of work (31%), which differed from Canadian respondents, who most often reported injuries only, or were treated by a doctor only (both at 16%). While the US respondents reported significantly more injury time loss claims than Canadian respondents (*p* = 0.025), similarly high proportions reported having returned to work.

**Table 5:** Characteristics of work-related injuries among active female firefighters in the US and Canada, by country.

	**Entire US sample (*n* = 772)**	**Entire Canada sample (*n* = 388)**	
	**Median (Q1, Q3)**	**Median (Q1, Q3)**	***p*** **-value (α = 0.05)**
Number time loss claims/respondent	0 (0, 1)	0 (0, 0)	**0.015**
Average time loss/injured respondent (months)	1.5 (0.5, 4)	1 (0.3, 3)	**0.012**
	***N*** **(%)**	***N*** **(%)**	***p*** **-value (α = 0.05)**
Injured at work	554 (72)	188 (48)	**<0.001**
**Injury severity** ^a^			**0.012**
Reported only	178 (23)	64 (16)	–
First aid only	117 (15)	53 (14)	–
Treated by doctor only	185 (24)	64 (16)	–
<2 weeks lost work	134 (17)	48 (12)	–
2–4 weeks lost work	77 (10)	29 (7)	–
>4 weeks lost work	238 (31)	47 (12)	–
Life-threatening injury	13 (2)	^*^	–
Injury time loss claim	242 (31)	97 (25)	**0.025**
Returned to work (*n* = 242, 97)	228 (94)	90 (93)	0.621
	**US injury time-loss claims (*****n*** **= 242)**	**Canada injury time-loss claims (*****n*** **= 97)**	
	***N*** **(%)**	***N*** **(%)**	***p*** **-value (α = 0.05)**
**Event during injury**			0.190
Fire ground	71 (29)	26 (27)	–
Training	54 (22)	14 (14)	–
Non-fire emergency	46 (19)	17 (18)	–
In-transit	10 (4)	8 (8)	–
Other on-duty	61 (25)	32 (33)	–
Protective equipment worn while injured	161 (67)	72 (74)	0.167
Pre-existing condition contributed to injury	14 (6)	12 (12)	**0.039**
**Injury type** ^a^			0.074
Strain/sprain/muscle pain	129 (53)	59 (61)	–
Dislocation/fracture	57 (24)	18 (19)	–
Wound/cut/bleeding/bruise	19 (8)	11 (11)	–
Other respiratory distress	7 (3)	^*^	–
Eye injury	7 (3)	0 (0)	–
Smoke/gas inhalation	^*^	^*^	–
Thermal stress	^*^	^*^	–
Fire/chemical burn	^*^	0 (0)	–
Act of violence	^*^	^*^	–
Other injury	72 (30)	21 (22)	–
**Injured body part** ^a^			0.589
Extremity	73 (30)	21 (22)	–
Back	60 (25)	23 (24)	–
Shoulder/chest	52 (21)	16 (16)	–
Hand	29 (12)	13 (13)	–
Foot	22 (9)	9 (9)	–
Head/face	15 (6)	10 (10)	–
Abdomen	^*^	^*^	–
Other	33 (14)	18 (19)	–
**Contributing factor** ^a^			**0.017**
Human error	53 (22)	23 (24)	–
Firefighter fatigue	24 (10)	10 (10)	–
Lack of situational awareness	17 (7)	14 (14)	–
Weather/act of nature	16 (7)	14 (14)	–
Decision making	18 (7)	10 (10)	–
Equipment failure	15 (6)	10 (10)	–
Lack of communication	14 (8)	8 (8)	–
Crew size	16 (7)	^*^	–
Lack of wellness/fitness	13 (5)	^*^	–
Lack of teamwork	11 (5)	^*^	–
Officer/incident command	6 (2)	5 (5)	–
Dangerous substance	8 (3)	^*^	–
Civilian error	7 (3)	^*^	–
Lack of training	5 (2)	^*^	–
Structural failure	5 (2)	^*^	–
Act of violence	^*^	^*^	–
Standard operating guideline/procedure breach	0 (0)	5 (5)	**–**
Protective equipment not worn	^*^	^*^	–
Protocol breach	0 (0)	^*^	–
Horseplay	0 (0)	^*^	–
**Return to work schedule** ^a^			**0.018**
Modified duties	81 (33)	37 (38)	–
Graduated return	81 (33)	19 (20)	–
Other supports	38 (16)	24 (25)	–
New policy made directly resulting from injury	20 (8)	10 (10)	0.549

a *Questions in which participants could select multiple answers*.

* *Fewer than 5 respondents*.

*Bolded p-values represent statistically significant differences (p ≤ 0.05)*.

Generally, characteristics of work-related injuries were similar among respondents in the US and Canada, however a significantly higher proportion of injury time loss claims in Canada reported pre-existing conditions that contributed to the injury (*p* = 0.039). Significant differences between the US and Canada were also found for contributing factors (*p* = 0.017), and return to work schedules (*p* = 0.018).

## Discussion

This study surveyed 1,160 active female firefighters in the US and Canada to address knowledge gaps and provide information about female firefighter injuries. Overall, our sample was similar to that described by Jahnke et al. study (18), with the average age of our injured respondents being 42.5 (SD 9.1) years compared to 38.8 (SD 9.2), and having an average of 16.1 (SD 8.8) years in the fire service as compared to 14.4 (SD 8.6).

While it has been reported that female firefighters in North America suffer more injuries than in other regions (12), direct comparisons between the US and Canada are lacking. We identified a higher proportion of female firefighters in the US reporting injuries than in Canada. Among those who had been injured, the proportion of injuries among US vs. Canadian respondents increased with higher injury severity. Similar to our findings, the US had a greater number of work-related injuries among firefighters from 2010 to 2014 as compared with 32 other countries (19). Our data also indicate that female firefighters in the US were older, had a higher BMI, and had a greater number of career fires and toxic exposures than those in Canada, and, on average, served in the fire service longer than respondents in Canada. All of this points to the increased risk of injury with more exposures to high risk situations; older, more experienced firefighters have been involved in more emergency responses.

Other studies have identified that firefighters with a higher-than-normal BMI were three times more likely to file an injury-related compensation claim compared with those of normal BMI (20). Higher overall good self-rated health, fitness, and exercise have all related to a lower likelihood of sustaining an injury (13). Low fitness levels among firefighters can increase injury risk (20), and compromise occupational readiness. Similarly, low fitness among women entering the US Army, as indicated by a failed 5-min step test, experienced more stress fractures and musculoskeletal injuries early in their careers as compared with more fit individuals (21). Programs promoting wellbeing among firefighters, including injury prevention and rehabilitation components, have resulted in significantly fewer workers' compensation claims (22), and well-designed exercise interventions have been shown to enhance overall firefighter health and fitness (23).

A slightly higher proportion of injured respondents reported tobacco use and alcohol consumption than those not injured. Previous research found that 16.5% of female firefighters who consume alcohol were problem drinkers, and that problem drinkers were 40% more likely to report work-related injuries compared with the general female firefighter population (24). Female firefighters with a history of tobacco use have also been found to have higher odds of injury (13).

In both countries, career firefighters and those assigned to an engine made up the largest proportion of injuries. Similarly, studies have found that career firefighters in the US were injured on the job more than volunteers, although these samples were predominantly males (3, 4). In the US and Canada, injuries occurred most often on the fire ground, adding to the conflicting literature describing firefighter injuries, some of which report training as the most common activity at the time of injury (11, 13, 18), and some of which report firefighting, fire ground duties, or fire station duties as the most common activities at the time of injury (4, 18, 25). In this study, training was the second most commonly reported event at the time of injury, supporting the evidence that the fire ground and training both present a high risk of injury for female firefighters; safety improvement efforts should be focused there.

In terms of injury severity, fireground firefighters made up a smaller proportion of injured respondents as severity increased, while company/station officers made up a larger proportion. Although there is little existing information regarding rank and injury severity, our findings concur with studies on predominately male firefighters. The first found firefighter/paramedics made up a larger proportion of those who had been injured than those who had not been injured (18), while the second found that company officers had one of the lowest proportions of injuries (11). We found that, other than master technicians for which all eight respondents reported an injury, company/station officers reported the highest frequency of injuries, however, our cross-sectional data cannot confirm whether respondents held these ranks when injured.

Men and women typically have different body shapes, sizes, and compositions, yet only 25% of female firefighters previously surveyed in North America had access to female-specific personal protective equipment (12). This may have contributed to our findings that 69% of career and 80% of volunteer female firefighters were injured despite the use of protective equipment during the injury event. Ill-fitting equipment is insufficient in protecting the female firefighters against injury, by impairing movement and reducing agility. Thus, the use of female-specific protective equipment is required for optimal performance and injury reduction.

Strains/sprains/muscle pain were the most common injuries identified, followed by dislocations/fractures, and wounds/cuts/bleeds/bruises. This is similar to another sample of female firefighters from the US, who reported predominantly dislocations/strains/sprains, followed by superficial injuries/open wounds (13). Other injuries, including eye injuries or smoke/gas inhalation, occurred in lower proportions, potentially because protective equipment reduced the risk for these injuries. The extremities were most often injured, followed by backs, and shoulders/chests. This differs from previous research on female firefighters that reported the most frequent involvement of the abdomen, pelvis, or back (including lower back, lumbar spine) (13, 26, 27). However, it is difficult to compare results between studies, because the extremities option in this study was broader, capturing more injures than other studies that often break options down as foot, lower leg, upper thigh, etc.

Overall, human error was the most frequently reported contributing factor, followed by firefighter fatigue. Reform is needed for female firefighters to be able to work safely; appropriate protocols are needed to prevent overexertion that can lead to injury. These findings also emphasize that good health and adherence to female-specific safety practices at the individual and organizational level are imperative. Focused efforts in this area will reduce strain on the fire service in terms of chronic injury and time loss claims.

While historically male-dominated professions in the US and Canada, such as physicians, are seeing a greater proportion of women in the workforce over time (28, 29), firefighting has experienced much slower recruitment of women. This is reflected in how female firefighters view their occupation and their role within it. Canadian female firefighters reported feeling “othered” due to discrimination and hostility in the workplace, leading to self-doubt (30). Female firefighters in Ontario discussed challenges in performing physically demanding job duties compared with male colleagues, which caused them to implement creative solutions to overcome obstacles, as requests for assistance were often linked with negative male attitudes (9). This challenging work environment likely contributes to injuries among female firefighters, and is an important female-specific issue in the firefighting profession. Even though female firefighter-specific challenges have previously been reported, of the 339 respondents who reported an injury-time loss claim in the present study, only 30 (9%) reported that a new policy succeeded their injury.

Our survey results indicate that work-related injuries are a significant issue among female firefighters in the US and Canada. We identified key factors involved in such injuries, as well as the circumstances surrounding these events, to arm stakeholders with additional information to aid in developing protocols and policies to reduce injury risk for female firefighters.

Given the nature of the present study, there are limitations. As the data were self-reported, accuracy of the responses cannot be verified. In some instances, respondents may not have felt comfortable answering questions honestly, which could bias the results. Since this survey was promoted as a work-related injury and cancer survey, respondents who were involved in such events may have been more or less inclined to complete the survey, which can decrease generalizability of the results. Further, as this study only captured an estimated 0.8% of female firefighters in the US and 5.8% in Canada, results may not be representative of the entire population of female firefighters in either country. Additionally, as minor injuries, such as bruises and cuts, are relatively common, they may have been under-reported due to recall bias. Conversely, female firefighters who were severely injured (or deceased) may not have been captured in this study. Finally, there are limitations with exploring the data by injury severity; time missed from work might be influenced by available length of paid leave, ability to take unpaid leave, jurisdictional differences in the availability of various healthcare services, or other factors.

Moving forward, future studies could test hypotheses about potential relationships between injury and variables not yet been analyzed. For example, subsequent research may explore the timing of when injuries occur along the career trajectory. Examining incidence, severity, location, and temporal aspects of work-related injuries by service function and gender would provide helpful information to directly compare the scope of injuries occurring among females compared with males.

## Data Availability Statement

Due to the ethical confidentiality restrictions, data are available upon request.

## Ethics Statement

This study was approved by the University of British Columbia Children's and Women's Research Ethics Board (H18-03318). Survey respondents provided informed consent at the start of the on-line survey.

## Author Contributions

KK, LG, and IP: conceptualization. KT: investigation. SP, AZ, AP, and AW: data curation. SP: writing—original draft and visualization. IP: supervision. All authors: writing—review and editing. All authors contributed to the article and approved the submitted version.

## Funding

This work was supported by City of Surrey Fire Department (grant number F19-00432).

## Conflict of Interest

The authors declare that the research was conducted in the absence of any commercial or financial relationships that could be construed as a potential conflict of interest.

## Publisher's Note

All claims expressed in this article are solely those of the authors and do not necessarily represent those of their affiliated organizations, or those of the publisher, the editors and the reviewers. Any product that may be evaluated in this article, or claim that may be made by its manufacturer, is not guaranteed or endorsed by the publisher.
